# Medical students’ perception of mobile learning during COVID-19 in Iran: A national study

**DOI:** 10.1371/journal.pone.0308248

**Published:** 2024-10-18

**Authors:** Soleiman Ahmady, Nasrin Khajeali, Noushin Kohan, Afagh Zarei, Bikram Biswas, Mohammad Barzegar, Azadeh Kordestani Moghaddam

**Affiliations:** 1 Professor of Medical Education, School of Medical Education and Learning Technology, Shahid Beheshti University of Medical Sciences, Tehran, Iran; 2 Department of LIME, Research Affiliated Faculty, Karolinska Institute, Solna, Sweden; 3 Medical Education Department, Ahvaz Jundishapur University of Medical Sciences, Ahvaz, Iran; 4 Department of Medical Education, Smart University of Medical Sciences, Tehran, Iran; 5 Educational Development Center, Birjand University of Medical Sciences, Birjand, Iran; 6 Department of Educational Administration, Noakhali Science and Technology, Noakhali, Bangladesh; 7 Department of English Language, School of Health Management and Information Sciences Iran University of Medical Sciences, Tehran, Iran; 8 University of Social Welfare and Rehabilitation Sciences, Tehran, Iran; Ahvaz Jundishapur University: Ahvaz Jondishapour University of Medical Sciences, ISLAMIC REPUBLIC OF IRAN

## Abstract

**Introduction:**

Mobile learning has gained significant attention in medical education in recent years. The COVID-19 crisis has further accelerated its adoption. A lack of research on student perceptions of mobile learning during pandemics limits strategies for maintaining education during these times. This study examines the perceptions of medical students in Iran regarding the mobile learning during COVID-19. It is imperative that these perceptions are understood to optimize mobile learning effectiveness in medical education during disruptions.

**Method:**

A cross-sectional study was done in 2022 among 785 medical students in Iran who spent summer semester. Convenience sampling was used to select the sample. We used Biswas et al.’s scale for measuring medical students’ perceptions of mobile learning during pandemics. Face and content validity was determined by qualitative methods. Internal consistency was measured with Cronbach’s Alpha (0.79). Data was collected through an online questionnaire. To analyze the data, descriptive and analytical statistics were conducted with SPSS software at a significance level of p<0.05.

**Results:**

In total, 1,200 medical students were asked to complete the survey, and 785 responded, resulting in a 65.4% response rate. Mobile learning has been embraced by majority of medical students, with Android devices being used the most frequently. They also have frequent access to the internet, and they rely on a wide range of apps and platforms for academic purposes. Students perceive mobile devices to be highly advantageous for improving subject knowledge (Mean = 4.71±0.58), accessing study materials (Mean = 4.44±0.75), and providing flexible learning opportunities (Mean = 4.40±0.79). Despite this, participants were less confident about the ability of mobile devices to assist with specific study problems (Mean = 3.12 ± 1.28), facilitate class discussions (Mean = 3.33 ± 1.38), and overcome screen size limitations (Mean = 3.32 ± 1.38).

**Conclusion:**

Medical students in Iran have widely adopted mobile learning and perceive it as beneficial for acquiring knowledge, accessing material, and being flexible during COVID-19. M-learning’s effectiveness in specific learning activities must be investigated in further research, and concerns regarding problem-solving, discussion facilitation, and screen size limitations should be addressed.

## Introduction

In the medical education sector, COVID-19’s unexpected arrival sparked a global earthquake [1]. Mobile learning (mLearning) arose as a beacon of hope due to the widespread use of smartphones and tablets, which provide unprecedented access to education resources [2]. A system that can be customized to meet each individual’s needs and accessed from any location and at any time [3].

mLearning has significant potential to improve medical students’ clinical decision making. It provides them with access to critical information at the point of care, enabling them to make informed decisions based on the latest evidence. It also provides them with practical applications and collaborative learning, interactive simulations, and real-time communication tools [4]. As a result, medical students will be able to make better clinical judgments and decisions. In addition, they will receive a tailored learning experience that meets their individual needs [5].

Although the rapid shift to mLearning revealed significant flaws, the existing curricula, teaching methods, and skillsets of educators and students were not sufficient to meet the demands of a mobile-centric, remote learning environment [6]. Also it was necessary to substantially revise the technical infrastructure to ensure proper operation [7].

Recently, mobile learning has gained popularity in Iran as a means of improving education opportunities and facilitating easy access to educational resources. As the mobile internet infrastructure grows, favorable conditions are being created due to the rapid rise of smartphones [7, 8].

In several studies, Iranian students’ perceptions and attitudes toward mobile learning have been examined providing an overview of both the benefits and challenges of its implementation, including enhancing student-teacher interaction, learning flexibility, and access to educational content [9–11]. Although mobile learning is a crucial component of medical students’ clinical training and decision-making skills, not enough research has been conducted to understand their perspective and needs, particularly during disruptive events such as the COVID-19 pandemic.

In the context of the Iranian medical education system, which traditionally relied on lecture-based instruction, the rapid shift to mLearning platforms during the pandemic presents a fascinating case study. While it was necessary to minimize disruption to the educational process, questions remained regarding their long-term viability and optimization potential. In order to develop future mLearning models, it is necessary to understand these limitations and analyze the user experience.

In this study, we examine Iranian medical students’ perceptions of mLearning during COVID-19 by investigating their perceptions of the resources, interactions, and challenges they faced. In this way, we can provide insights into Iranian medical education beyond the pandemic. We can also help shape best practices for developing a formal mLearning program in Iran.

## Background

Mobile learning (mLearning) has gained popularity in recent years [12]. In particular, during the COVID-19 pandemic, it played a key role in supporting medical education continuity [13].

Mobile devices such as smartphones, e-books, tablets, and iPads facilitate electronic-mediated teaching and learning. By leveraging extensive communication capabilities, these tools facilitate collaboration between geographically dispersed learners [14].

Mobile learning is defined as the use of mobile/wireless technologies to deliver educational content. Taking into account technological, learner, and contextual mobility factors, flexible learning takes place across a variety of environments [15].

Mobile learning also refers to the use of mobile devices to access curricula anytime, anywhere. Students’ diverse learning preferences and schedules are accommodated through this pedagogical flexibility [16].

In recent years, mLearning has revolutionized teaching methods in medical education by enabling self-directed and adaptive learning to occur. Accessing the curriculum from multiple devices is possible through wireless connectivity. Unlike e-learning, which is asynchronously delivered through online networks, mLearning is accessed remotely [17, 18].The widespread use of mobile learning and the availability of current and relevant content, in addition to pedagogical innovations, make digital instruction a viable alternative to traditional teaching [18].

By exploring emerging andragogical designs through novel technologies, such as mLearning, it is possible to cultivate sustained high-quality medical education pedagogy. As a result, the standard curriculum can be systematically and interdepartmentally enhanced to achieve targeted competencies, such as autonomy and clinical decision making competencies [9].

Mobile learning is becoming increasingly popular in medical education. Several studies have examined the effectiveness of this method of teaching and learning in medical education. This is especially useful for providing clinical skills training and accessing clinical references on the go. Interactive learning approaches are essential for skilled physicians [19–21].

Mobile learning in Iran has shown great promise for improving medical education access. Mobile learning has become an effective method of delivering curriculum inside and outside the classroom. This is due to the widespread use of smartphones and tablets. It can facilitate self-directed study of clinical and theoretical topics when fully integrated into pedagogy, thereby providing educational resources at all times and from any location to Iran’s large student population. Mobile apps and platforms have evolved into an effective complement to traditional teaching methods. Medical institutions in Iran have adopted M-learning strategies to take advantage of widespread mobile device use among students. Students tend to engage more deeply with interactive learning activities and feedback systems via mobile interfaces than passive lectures due to their interactive nature. In order to maximize the benefits of the program, students living in remote or rural areas must have access. In addition, educators must be trained on multimedia course materials design [22, 23].

Due to technology advancements, mobile learning holds significant potential for enhancing competency-based, collaborative models emphasized in Iran’s medical reforms. Researchers are exploring ways to optimize integration by balancing innovative practices with sociocultural considerations as part of this new field of study [9, 24].

## Methods

A cross-sectional study was done in 2022 among medical students in Iran.

### Setting

This study was conducted at Smart University of Medical Sciences, one of Iran’s top health sciences universities. This university, located in Tehran, was founded in 2015.

### Participants

Medical students from across Iran participated in the summer semester at Smart University, the country’s national hub for virtual education. We included in our study medical students in Iran who are currently enrolled in medical schools, possess a mobile device, and are willing to take part. In addition to students without access to mobile devices, those who declined to participate were not eligible to take part.

A convenience sampling method was used in this study. The questionnaire was distributed to 1,200 students, with a final participation rate of 785 (65.4%). A potential non-response bias resulting from the COVID-19 pandemic and the limitations of online questionnaires was partially mitigated by extending the data collection period.

### Instrument

This study used Biswas et al.’s instrument. They developed this scale for assessing medical students’ perception of mobile learning during the Bangladesh pandemic [2020]. The questionnaire consists of three parts. Part 1 gathers primary information about students and information about the information technology of students’ cell phones. It also gathers information about Internet access times, smartphone access times every day, whether they use mobile phones for academic goals or not, and excessive use of social media. The second part examines the use and preference for social networks for education and interaction with social media applications. Students’ perceptions and attitudes regarding mobile learning and social media learning tools during the COVID-19 crisis are assessed in part 3 of the questionnaire.

The questions of the third part included a 5-point Likert scale ranging from ’Strongly Disagree’ (1) to ’Strongly Agree’ (5), and the students’ perceptions of mobile learning were operationally defined as positive (mean score ≥ 4.0, indicating strong agreement with positive statements), relatively positive (mean score ≥ 3.5 and < 4.0, indicating agreement with positive statements but not as strongly), and negative (mean score < 3.5).

In Bangladesh, this scale is validated and tested on a sample population of Bangladeshi students. We followed WHO guidelines for translation and cultural adaptation. During the face validity assessment, eight experts evaluated the questionnaire’s appearance and format in terms of its clarity, simplicity, and relevance to the study objective. For content validity, the same eight experts examined whether the questionnaire content adequately covered the intended concepts and whether the items accurately reflected them. Based on the qualitative review conducted by experts, the questionnaire was found to be valid from a face and content validity perspective. This indicates a well-designed instrument. Cronbach’s test measured the degree of internal consistency (0.79).

### Procedures and data analysis

A web-based survey was carried out. Surveys were conducted anonymously using Google Forms software. The study purpose was explained to medical students and permission to participate was sought. They were asked to provide information about demographics including gender, age, and academic year levels. Data analysis was done using the statistical software package IBM SPSS Statistics V 22 and a p-value of less than 0.05 was determined significant. Descriptive statistics, such as means, standard deviations, frequency counts, and percentage distributions, were generated.

The Kolmogorov-Smirnov test was performed to check the data normality. Furthermore, the T-test was used to evaluate age and gender differences, and variance analysis was performed to determine academic year levels.

### Ethical considerations

Smart University of Medical Sciences approved this research through the research ethics committee with the registration number IR.VUMS.REC.1401.022. Written informed consent was obtained from all participants, and they were assured of anonymity and confidentiality throughout the study. Participants were protected from risks and discomfort through the study design.

## Results

In total, 1,200 medical students were asked to complete the survey, and 785 responded, resulting in a 65.4% response rate. Despite a relatively high response rate, there were significant non-responses, which is a common issue when collecting data online and pandemic limitation. While the researchers made every effort to increase participation, such as sending multiple reminders and extending the data collection period some degree of non-response was inevitable.

The sample included 302 males (38.47%) and 483 females (61.53%). Table 1 shows the demographic data of the respondents. As shown in Table 2, 785 medical students were distributed across the medical universities in Iran. This study examines medical students’ perceptions of mobile learning during the pandemic. Table 3 shows section results As seen in Table 4, the t-test indicated a statistically significant difference between genders (t = 5.17, p < 0.005), and mobile learning. Analysis of variance indicated a statistically significant difference between average grades of different medical school years (F = 3.02, p < 0.05).

**Table 1:** Demographic data of the respondents.

Group	N
Gender	
Male	302
Female	483
Age	
< 23 Years old	502
> 23 Years Old	283
Academic Year	
1st year	102
2nd year	155
3rd year	124
4th year	101
5th year	77
6th year	114
7th year	112

**Table 2:** Participants in the Iranian Medical University’s research.

**1**	**University**	**Number of Students**	**Percentage**
**2**	Tehran University of Medical Sciences	157	20%
**3**	Shahid Beheshti University of Medical Sciences	118	15%
**4**	Isfahan University of Medical Sciences	94	12%
**5**	Shiraz University of Medical Sciences	79	10%
**6**	Mashhad University of Medical Sciences	71	9%
**7**	Iran University of Medical Sciences	63	8%
**8**	Kerman University of Medical Sciences	47	6%
**9**	Tabriz University of Medical Sciences	39	5%
**10**	Ahvaz Jundishapur University of Medical Sciences	39	5%
**11**	Other Medical Universities	78	10%
**12**	Total	785	100%

**Table 3:** Medical students perception of mobile learning during Covid-19 pandemic.

Questions	Mean ±SD	Perception level
Mobile learning is flexible to learn anytime, anywhere.	4.40±0.79	Positive
During the Covid-19 pandemic, mobile learning is an effective way to minimize the study gap.	3.83±1.33	Relatively Positive
The mobile app makes it easier for me to find relevant information about my studies.	4.30±1.01	Positive
Mobile learning improves my study skills.	3.84±1.26	Relatively Positive
Mobile devices make it easier to access study materials.	4.44±0.75	Positive
The use of mobile devices makes it easier to share in class-related discussions both online and offline during the covid-19 period.	3.33±1.38	Relatively Positive
The use of mobile devices helps me to improve my knowledge in my field of study.	4.71±0.58	Positive
Mobile learning helps to develop my motivation to finish my studies during this pandemic time.	4.13±1.26	Positive
Mobile devices help me solve study problems.	3.12±1.28	Relatively Positive
Social media applications help educational fulfillment during the covid-19 period.	4.40±0.79	Positive
Social media helps to strengthen communication with others	4.10±1.11	Positive
It is faster to get feedback with mobile learning.	4.24±1.26	Positive
Mobile learning improves interactivity between student and teacher.	3.33±1.38	Relatively Positive
During COVID-19, mobile devices serve as a companion to learning.	4.17±1.04	Positive
High mobile internet charges during pandemic may have an impact on my academic performance.	4.10±1.11	Positive
I think mobile learning will help me to recover my study gap at this pandemic time.	4.40±0.7	Positive
Screen size of my mobile does not affect my learning.	3.32±1.38	Relatively Positive

**Table 4:** Comparison of mean mobile learning perception scores by gender, age, and academic year.

Group	N	Mean	SD	Statistical Significance
Gender				
Male	177	106.29	13.53	p < 0.05
Female	376	111.53	10.72	t = 5.17
Age				
< 23 Years old	312	114.01	13.08	p = 0.20
> 23 Years Old	241	112.63	13.94	t = 1.17
Academic year				
1st year	89	107.36	13.35	p < 0.05
2nd year	78	109.19	11.39	F = 3.02
3rd year	101	110.50	10.80	
4th year	99	112.12	13.55	
5th year	60	114.01	13.08	
6th year	76	106.29	13.53	
7th year	50	108.19	11.39	

This score reflects the average perception of mobile learning for each group using a five-point Likert scale.

More than 87% of students’ mobile phones use the Android operating system and the rest are utilizing iOS (11.0%), Color OS (1.0%), Windows (1.0%), and others (0%). 78.5% of medical students always access the Internet to search for information, 21.3% always access the Internet, 12.6% of students utilize the Internet occasionally and 2.7% very infrequently. 70% of students use mobile phones for learning purposes. About 87.7% of students utilize mobile phones between 1 and 5 hours of their time, while about 12.2% of students spend more than 7 hours on mobile phones. About 43.0% of medical students prefer WhatsApp for learning, 30% to Google, 15.5% to YouTube, 10.2% to Zoom, and 33.2% to Telegram.

## Discussion

Medical students increasingly utilize smartphones and apps to facilitate interaction, communication, and collaboration with their classmates. In addition, they can learn anywhere. Most health team professionals and medical students utilize mobile devices to communicate with patients (e-mail, telephone, text), and often use them daily. This is especially true when faced with unpredictable conditions such as the COVID-19 pandemic. Medical students reported using smartphone medical apps as their first reference. Medical students use clinical references, drug references, diagnosis aids, and clinical calculators [25, 26].

The present study showed that more than 87% of medical students use Android phones. The remaining are iOS (11.0%), Color OS (1.0%), Windows (1.0%), and others (0%). However, this result was previously described. Gavali et al. In 2017, it was found that most medical students use smartphones. After that, Android, iPhone, Windows-based Nokia, and Blackberry are priorities [27]. In Singh et al. Apple’s iPhone (55.4 percent) was the most used brand in 2021, followed by Google’s Android (43.8 percent) [28].

In the study of Rohilla et al 2021, Out of 222 participants, 198 (89.2%) Android devices had 13 (5.7 percent) iPhones, followed by other operating systems including Windows and Blackberry 11 (4.9 percent) [29]. These findings further support the idea that four factors drive the use of various types of smartphones: cost, convenience, coverage, and utility [30]. The more students know about the application, usefulness and perceived ease of technology, they will have a better attitude towards it. They will show a greater behavioral tendency to utilize it. In this way, users will start using technology for real.

Another finding was that 99.8% of medical students use the Internet almost always, and 2.1% rarely. These findings are consistent with Ayatollahi et al.’s study (2014), which shows that medical students refer to access medical-related topics, with the highest percentage of referrals being to the PubMed database and specialized journals. A large number of students (78.9%) use the Internet to access articles, clinical photos and radiographs, and educational videos [31].

According to this study, 70% of respondents use mobile phones for academic purposes. Dias et al. (2022) showed that university students widely utilize mobile phones for educational activities and consider them valuable educational tools [32]. In a study by Milheim et al. (2021), learners utilize mobile devices for online courses or course-related activities, such as note-taking and studying course material. Most respondents to this study use mobile devices because they are convenient, portable, and easy to utilize [33]. Due to the use of various capabilities and benefits of mobile phones, students can receive more interesting and diverse content. This causes an increase in motivation and attitude.

Khan’s (2021) findings confirm that all participants (n = 270) had smartphones. They used smartphones to search the Internet for medical questions (100%), share educational resources (90%), and take notes (79%) [34]. Among the possible reasons for the increase in students’ attitude towards learning through mobile phones are practice and repetition with time intervals and timely feedback and the use of several comprehensive senses, the availability of this device without time and place limitations, and the ability of this device to be multimedia. Making communication between the learner and the teacher easier than in the traditional education environment. This is done by using more time to connect the informal learning environment to the formal one. It also establishes the flexibility of this method compared to traditional learning methods, and establishing easier and pressure-free communication with the teacher [35].

About 87.7% of students use mobile phones between 1 and 5 hours of their time, while about 12.2% of students spend more than 7 hours on mobile phones. A similar study of Bangladeshi students found that mobile phones were used 3–4 hours a day [36]. Similarly, Siddiqi et al (2017) found that 50% of medical students accessed the internet on their portable devices via Wi-Fi for more than 4 hours daily [37].

The use of different social networking sites and the ease of access to various social networking sites have made mobile devices more popular in recent years. For this reason, students, including those at the university level, take advantage of these mobile device opportunities through collaboration, interaction, and interaction. This allows them to easily share educational content using these social media platforms [38].

43% of medical students in our study prefer WhatsApp for studying, 32.3% to Telegram, 30% to Google, 15.5% to YouTube, and 10.2% to Zoom. Various studies have shown that students demand academic applications to support learning [39–41]. Social media apps also play an important role in this regard. Perhaps their access to social media is better.

The majority of medical students in our study have a positive attitude about mobile learning relating to the flexibility to learn anytime, anywhere. This is because they can find relevant information, access study materials, improve cognition in the area of study, cause high motivation to finish their studies, and solve study-related problems. It is a good idea to use social media programs to meet educational goals in the era of COVID-19, as it is a faster and more effective way of getting feedback; this is also a good opportunity to increase student-faculty interaction during this difficult time; and they believe that the mobile phone can serve as an educational aid in any situation. This can be very useful in an unpredictable situation like COVID-19. They believed that my smartphone screen size did not affect their learning. In general, medical students in our study had a positive attitude towards mobile learning during Corona (Table 2).

This study has results that are in line with previous works in this field such as Al-Hijri (2017), Hussein and Ahmad (2016), Rahman (2014), etc [38, 41, 42]. Also, Wallace et al. It was discovered that mobile devices enhanced students’ perception of academic efficiency [43]. Mahmoudi et al (2014), showed in their study that teaching through mobile phones, such as lectures, promotes students’ learning and memory [44].

A statistically significant relationship exists between students’ academic years and gender and mobile learning use, according to a comparison of group scores. (Table 3). Gender or age can also affect a technology’s acceptance level [45]. Dutch behavior is studied online. The researchers found that gender and age impact Internet use. Internet use is more prevalent among males and young people. Males are more adept at using technology than females, for example, and have less anxiety about it [46]. Age plays a significant factor in social networking site usage. According to their findings, younger students visited social networking sites more frequently than older students [47].

Another point, Mobile learning has the potential to significantly impact medical students’ clinical skills, including diagnostic reasoning and patient communication. Such as access to clinical resources: Mobile learning platforms provide medical students with instant access to a wide range of clinical resources, such as medical textbooks, research articles, clinical guidelines, and interactive atlases. This access to comprehensive and up-to-date information enhances students’ diagnostic reasoning abilities by enabling them to quickly retrieve relevant information and make informed clinical decisions. Interactive case studies and simulations: Mobile learning applications can offer interactive case studies and clinical simulations that allow students to practice their diagnostic skills in a controlled environment [37].

This study was limited in that it was based on a sample from a single country. This limited its generalizability to other contexts. It would be advantageous for future research to adopt a more comprehensive sampling approach in order to gain perspectives from a wide range of cultural and geographical backgrounds. It would be possible to conduct cross-cultural comparisons if, for example, universities collaborated with international universities or recruited international students on a purposeful basis.

Additionally, the study focused exclusively on student perceptions without taking into account the viewpoints of other stakeholders, such as educators and administrators. Multi-perspective approaches contribute to a better understanding of the subject matter because they examine a variety of experiences. For insights into implementing mobile learning, researchers could administer surveys or conduct interviews with instructors, support staff, and management.

Furthermore, the specific context of mobile learning was not taken into account, which may have influenced responses. For future studies, a standardized environment or platform could be utilized in order to mitigate potential confounding factors. It may be possible, for example, to provide all participants with access to curricular materials through one customized mobile application, thereby minimizing variation in experience.

In order to address these limitations, a more inclusive sampling strategy, multi-stakeholder data collection, and controlled learning environments would be essential to strengthen the validity and generalizability of findings. The results of this study will also assist in developing recommendations to better support mobile learning in medical education that are informed by diverse perspectives.

It is believed that the findings of this study will be of great importance for medical education in low- and middle-income countries, where mobile learning may be of particular value. When resources are limited, m-learning’s accessibility and flexibility can facilitate the extension of educational opportunities and make it possible to reach students in remote or underserved areas. According to the Iranian medical students, m-learning has the potential to be an effective instructional modality for similar settings in developing countries based on their positive perceptions of its usefulness, usability, and overall satisfaction. The design and implementation of mobile learning programs to support continuous education in low- and middle-income countries can be optimized by understanding the factors influencing student acceptance and adoption of mobile learning. This will be particularly valuable when disruptions such as the COVID-19 pandemic occur. Through the widespread use of mobile devices among students in these regions, geographical barriers can be overcome and equitable access to high-quality medical training can be enhanced.

## Conclusions

This study measured students’ perceptions of mobile learning to learn during the pandemic among Iranian medical students. This study showed that learners are knowledgeable about. Mobile learning, have a positive understanding of mobile learning and use social media. The findings show that mobile- learning provides learners with the opportunity to participate in classrooms from anywhere during pandemics. Future research can explore the impact of m-Learning on the acquisition of knowledge, attitudes, and skills of medical students in the COVID-19 and post-COVID eras, and its impact in theoretical and medical education.

Policymakers and educational institutions should consider incorporating mobile learning technology into the entire educational institution. Medical educators need to keep these factors in mind when designing mobile learning programs. One-size-fits-all mobile learning initiatives will not work.
